# Immediate help through group therapy for patients with somatic diseases and depressive or adjustment disorders in outpatient care: study protocol for a randomized controlled trial

**DOI:** 10.1186/s13063-015-0801-3

**Published:** 2015-06-27

**Authors:** Miriam Ruesch, Almut Wiebke Helmes, Juergen Bengel

**Affiliations:** Department of Rehabilitation Psychology and Psychotherapy, Institute of Psychology, Albert-Ludwigs-University Freiburg, Engelbergerstr. 41, 79085 Freiburg, Germany

**Keywords:** Health services research, Comorbid somatic and mental diseases, Depression, Adjustment disorder, Group psychotherapy

## Abstract

**Background:**

One in three people with a chronic somatic disease suffer from a comorbid mental disorder. Most common comorbidities are depressive, anxiety and adjustment disorders. These lead to an increase in morbidity and mortality, and a deterioration of quality of life and healthcare costs. Treatment of mental disorders is of great importance, but the waiting time for outpatient individual psychotherapy can be up to six months in Germany. Group therapy has comparable treatment effects and is considerably more economic than individual therapy; however, it is still almost unused in the outpatient care system. The introduction of a stepped care approach, such as attending a group program before individual therapy, could improve this issue. For this purpose we developed a group program (STEpS), and its efficacy will be evaluated in this study.

**Methods/Design:**

A randomized controlled trial will be conducted to evaluate the efficacy of a cognitive behavioral therapy (CBT) group program for patients with somatic diseases and depressive or adjustment disorders, compared to a wait-list control group. A total of 128 adults with any chronic somatic disease and comorbid depression or adjustment disorder will be recruited in our outpatient clinic, and will be randomly assigned to participate in the group program immediately after contacting the clinic (intervention group) or after a waiting period of four months (wait-list control group). Primary outcomes will be self-reported depressive and anxiety symptoms. Secondary outcomes will be self-reported psychological distress, changes in experience and behavior, health-related quality of life, state of self-esteem and subjective need for therapy. Assessments will take place at baseline, 10 weeks (post-treatment) and 18 weeks (follow-up) after randomization. Additionally, treatment acceptance and psychotherapeutic process will be assessed after each session.

**Discussion:**

This study investigates whether the CBT group program is an effective treatment to reduce depressive and anxiety symptoms and psychological distress in these patients. If the group program is effective, it could be implemented as a treatment option prior to individual outpatient therapy. These results will contribute to improving outpatient care for mental disorders in patients with somatic diseases.

**Trial registration:**

German Clinical Trials Register DRKS00005140 (27 August 2013).

## Background

Chronic somatic diseases are an increasing health problem in modern society. Due to improved treatment options, an ageing population, and harmful health behavior and lifestyles, the incidence of chronic somatic diseases, such as cardiovascular diseases, stroke, cancer, chronic respiratory diseases and diabetes is increasing. According to recent estimates, 38.8 % of the German adult population have a chronic disease [1]. In the European Union, chronic diseases are responsible for 80 % of deaths and for 77 % of years lived disabled or lost due to premature death (known as disability-adjusted life years (DALYs)) [2, 3].

People with chronic diseases have to cope with many stressors, such as symptoms, treatment, role changes, relapse, uncertainty concerning the progression of their illness, and the threat of death. These patients have a significantly increased risk for developing mental disorders. Härter *et al*. [4] reported a 12-month prevalence rate of 42.5 % for mental disorders in patients with chronic somatic diseases. A physically healthy comparison group showed a significantly lower prevalence rate of 25 % (odds ratio: 2.2) for mental disorders. The prevalence rates of mental disorders do not differ significantly for patients with different somatic diseases [4]. The most common mental comorbidities with somatic diseases are affective disorders (12-month prevalence: 21.1 %) and anxiety disorders (12-month prevalence: 22.9 %) [4]. Although little epidemiologic data exists, adjustment disorders are among the most common mental disorders diagnosed in patients with chronic somatic diseases [5, 6]. In outpatient care, adjustment disorders are diagnosed very often: between 5 and 20 % of patients in outpatient care have an adjustment disorder as their main diagnosis [7]. Prevalence rates for adjustment disorders range from 7.1 % in patients with breast cancer [8] to 12 % in a consultation-liaison psychiatry service [9] and to 17 % in patients with cardiovascular diseases [10].

Comorbid mental disorders have negative effects on morbidity, mortality, quality of life and healthcare costs [11]. These negative effects underline the importance of psychotherapeutic care of mental disorders in patients with chronic somatic diseases. However, in Germany patients searching for outpatient psychotherapy have to be prepared for waiting times of several months. According to data from the German Federal Chamber of Psychotherapists, a patient waits on average 12.5 weeks for an initial consultation and 23.4 weeks for the beginning of an outpatient psychotherapy [12]. Long waiting periods increase patients’ dissatisfaction with the healthcare system [13], and are one reason why patients do not appear for their initial appointments [14, 15] or give up their intentions for psychotherapy [16]. These patients on a wait-list do not seek mental health services from other professionals [17]. Although approximately 23 % of cases of untreated depression remit spontaneously within three months, and 32 % in six months [18], long waiting times mean that patients who do need professional help to overcome their depression remain depressive for a longer period. Since patients with depressive disorders and also persons with subthreshold depressive symptoms have significantly more days of absence from work [19], the emerging indirect costs through long waiting times should also be considered.

One approach to bridge the gap between the demand and availability for outpatient psychotherapy is to increase efficiency of care through the adoption of a stepped care treatment. Stepped care approaches aim to provide minimal interventions as first-line treatments (such as self-help, bibliotherapy and computer-based interventions) in order to reserve more intensive and expensive treatments like outpatient individual therapy for those who did not, or very likely will not benefit from these minimal interventions [20].

Group therapy can be classified as a medium intensive treatment option in the stepped care model [21]. Group therapy has the advantage of being able to treat many patients simultaneously; therefore, the costs for group therapy are less than half the costs for individual therapy [22]. In depression care, group therapy has been shown to be more economic than individual therapy [23]. The effectiveness of group therapy has been proven in several meta-analyses [24], and in general, the established effects of group therapy were comparable to individual therapy [25]. For depression treatment it can be assumed that group therapy is marginally inferior to individual therapy in the short-term comparison at post-treatment. When comparing long-term effects, group therapy for depression reaches similar effects compared to individual therapy [26]. For patients with somatic diseases, group therapy is also regarded as an effective treatment. In a meta-analysis of studies across different somatic diseases, a moderate pre to post effect size of *d* = 0.49 was found [24]. Solid empirical evidence exists for group therapy for cancer and HIV patients [24]. In a meta-analysis of 47 higher quality studies, Sherman *et al*. [27] examined the effectiveness of group interventions for cancer and HIV patients concerning psychosocial outcomes. Sherman *et al*. found that structured CBT group interventions are preferable for the treatment of patients with HIV or early-stage cancer, whereas less structured and more existentially-oriented group interventions are preferable for patients with advanced cancer. Studies focusing on the effects of group therapy on comorbid depression or adjustment disorder in physically ill patients are relatively rare. The few existing studies report significant treatment effects but inconsistent effect sizes. Heckman *et al*. [28] reported a small effect size for a coping improvement group intervention (12 sessions) for patients with HIV and mild to severe depressive symptoms. Hambridge *et al*. [29] found a moderate pre to post effect of their CBT group program (six sessions) for patients with coronary artery disease and comorbid depression. Schuster *et al*. [30] found a strong effect for a CBT group therapy compared to treatment as usual for patients with coronary or orthopedic diseases and comorbid depression, anxiety or somatoform disorders.

Group therapy is very uncommon in outpatient care in Germany, with statistics from the National Association of Statutory Health Insurance Physicians (Kassenärztliche Bundesvereinigung) showing that group therapy represents only 1 % of invoiced psychotherapeutic services [31]. However, healthcare providers and directors of managed care organizations assume that the use of group treatments will increase in the future [32].

In depression care, cognitive behavioral group therapy has been recommended as a first-line treatment in stepped care [33]. According to the above-mentioned results, it can be assumed that in addition to individual therapy, group therapy could be a promising first-line outpatient treatment for patients with chronic somatic diseases and comorbid depressive or adjustment disorders.

### Study objective and research questions

This planned study aims to evaluate the efficacy of a CBT group program (STEpS) developed to provide immediate help before potential individual psychotherapy for patients with somatic diseases and comorbid depressive or adjustment disorders. We expect that self-reported depressive and anxiety symptoms assessed by the Hospital Anxiety and Depression Scale will be reduced to a greater extent in the intervention group compared to the wait-list control group. Furthermore, we hypothesize that the group difference in symptom severity can also be demonstrated two months after treatment, although the effect will be smaller. In addition, we expect that the intervention group will be superior compared to the wait-list control group in terms of self-reported psychological distress, changes in experience and behavior, health-related quality of life, state of self-esteem and subjective need for therapy. We also assume that the acceptance of treatment, assessed by attendance rates and self-reporting, is high. Session questionnaires should provide additional information about the psychotherapeutic process of the newly developed group therapy program.

## Methods/Design

### Study design

**Fig. 1:**
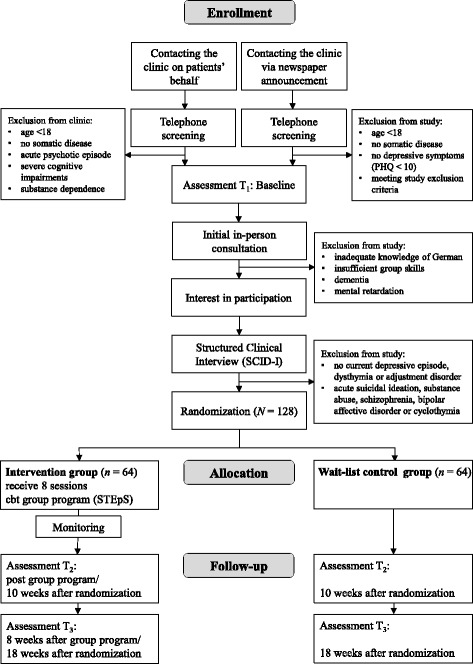
Study flow. *PHQ* Patient Health Questionnaire

This is a randomized, wait-list controlled trial. Study participants allocated at random to the intervention group will participate in the CBT group program for patients with somatic diseases and depressive or adjustment disorders immediately after contacting the clinic. Study participants allocated at random to the wait-list control group will follow the usual waiting process of our outpatient clinic. After completion of the follow-up assessment (about five months after randomization), study participants of the wait-list control group are also offered to participate in the group program. The three measurement points are scheduled for all study participants at baseline (immediately before initial consultation, T_1_), 10 weeks after randomization (post-treatment, T_2_) and 18 weeks after randomization (follow-up, T_3_). The 18-week follow-up measurement is scheduled in a relatively short interval of two months after treatment because we did not want to extend the usual waiting period of four months for individual psychotherapy for patients in the wait-list control group. For an overview of the study design see Fig. 1.

All study procedures are in line with the Consolidated Standards for Reporting Trials (CONSORT) 2010 statement [34] and the CONSORT Extensions for Reporting Pragmatic Trials [35]. The study protocol has been approved by the ethics committee of the Albert-Ludwigs-University of Freiburg (approval number 285/13), and is registered with the German Clinical Trials Register (identifier: DRKS00005140).

### Participants and procedure

Patients with any chronic somatic disease and comorbid depressive or adjustment disorder are the target group of this study.

#### Inclusion and exclusion criteria

Patients included in the study 1) are older than 18 years; 2) have a chronic somatic disease; 3) have a comorbid adjustment disorder, depressive episode (mild to severe, single or recurrent) or dysthymia, all according to the Diagnostic and Statistical Manual of Mental Disorders IV text revision (DSM-IV-TR) criteria; and 4) have sufficient skills to participate in a group setting (for example willingness for self-disclosure, sufficient communication skills and ability to adhere to group rules). This will be checked by clinical assessment in the initial consultation. We estimate that 95 % of the patients in our clinic will meet this last inclusion criterion.

Patients with acute suicidal ideation, comorbid substance abuse, schizophrenia, dementia, mental retardation, bipolar affective disorder, cyclothymia, ongoing psychotherapy or inadequate knowledge of German are excluded from the study.

#### Recruitment

Patients will be recruited after the initial consultation in our outpatient clinic for patients with somatic and mental diseases at the Albert-Ludwigs-University of Freiburg. In addition, we will send written information about the study to colleagues (such as general practitioners, medical specialists, psychotherapists and rehabilitation clinics) asking them to refer suitable patients to our outpatient clinic. Furthermore, we will deposit flyers in the waiting rooms of medical practices and will place announcements in the local newspaper.

#### Assessment of eligibility and randomization

There are two different approaches of admission: study participants may contact the outpatient clinic on their own behalf because they want to start psychotherapy, and study participants may also contact the clinic due to the newspaper announcement because they are interested in study participation. Patients who contact the clinic on their own behalf will have a preliminary short telephone screening by the head of the clinic. With this telephone screening we aim to check if patients generally fit with our clinic. Patients younger than 18 years, with no somatic disease, or who have an acute psychotic episode, severe cognitive impairments or substance dependence will be referred to other institutions. All other patients will undergo the further admission procedure of our clinic.

Study applicants who contact the clinic due to the newspaper announcement will also have a preliminary telephone screening. With this telephone screening, we aim to check if the study applicants fulfill the inclusion criteria and do not meet any of the exclusion criteria for the study. The telephone screening will be conducted by a trained clinician and includes a depression screening (Patient Health Questionnaire (PHQ-9) score ≥10 [36]), and questions about the somatic disease, alcohol, drug and medication consumption, schizophrenia (questions 8, 12, 13, 14 and 15 from the Early Recognition Inventory (ERIraos) checklist [37]), bipolar symptoms, possible difficulties in groups and current psychotherapeutic treatment. In cases of suspected substance abuse or bipolar disorder, these sections will be screened in more detail according to DSM-IV-TR criteria [38]. Dementia and mental retardation will be estimated by clinical assessment. Suitable applicants will undergo the further admission procedure of our clinic.

The following admission procedure is equivalent for both groups. Patients from both groups receive an appointment for an initial consultation within two weeks after the telephone screening. The initial consultation is conducted by the head of the outpatient clinic. If patients appear suitable for the study at the initial consultation, a research assistant will inform them about the study immediately after the consultation. Patients who are interested in participating will receive materials containing detailed information about the study’s content and procedures, and will be called within one week to schedule an appointment for a Structured Clinical Interview for DSM-IV Axis I Disorders (SCID-I) [39]. At the beginning of the SCID-I interview, we will inform participants about data security and that they may withdraw from the study at any time without any negative consequences (for example, without prolonging the waiting period for individual psychotherapy). Written informed consent will be obtained from all study participants. All SCID-I interviews will be conducted by trained clinicians and will be videotaped for quality assurance if patients give their written consent.

Patients who fulfill all inclusion criteria and none of the exclusion criteria will be randomly assigned to the study conditions. In order to guarantee a continuous group size in the STEpS group program, we decided to use block randomization with a block size of 20 and an allocation ratio of 1:1. The allocation sequence will be created by computer-generated random numbers, and will be carried out by a researcher who is not involved in the enrolment and allocation process. Staff involved in enrollment and allocation do not know the allocation sequence; we will use a sealed envelope system. Study participants will be informed about study exclusion or inclusion, allocation to study condition and further study procedures by telephone.

#### Blinding

In the planned study design it is not possible to blind study participants or therapists to group condition.

### Intervention

#### Intervention development

The group program was developed for the target group of patients with somatic diseases and comorbid adjustment or depressive disorders. The conditions of the group program were designed according to the following considerations:

1. In order to facilitate immediate access of patients to the group program after their initial consultation, the group structure needed to be open or half-open.
2. The waiting period of four months should not be prolonged, so the group program could only consist of eight sessions, also taking into account a follow-up measurement point.
3. Participants should receive psycho-education, and learn functional strategies to reduce psychological distress; therefore, the sessions required preset topics and a structured procedure.
4. The program should include different topics to satisfy the different problem areas of the patients.
5. Group size should be small or medium, in order to enhance social exchange and social support among patients.

The treatment manual for the STEpS group program was developed in four stages. First, we performed a systematic literature review of effective group therapies for patients with somatic diseases and comorbid adjustment or depressive disorders, and identified the most commonly used interventions from the effective group therapies. Based on these results, we developed a first draft of the group manual and ran a simulation using psychology students. Based on the students’ feedback we decided to dedicate two sessions to each topic, in order to enable the deeper elaboration of content within the different topics (for example assigning homework to be discussed in the second session). Thirdly, a team of experts made the final decision on the sessions’ topics and contents. Finally, four sessions of the final group manual were tested in a pilot group with three patients who met the inclusion criteria.

#### Intervention design

The STEpS group program consists of eight weekly sessions of 100 minutes. The group size will be limited to eight patients and the group setting will be half-open: entry for new members will be possible with every new topic (every other session). The group program will be performed by a psychotherapist trained in CBT with experience in group psychotherapy. Before joining the group, new group members will have a short consultation with the group therapist for about 20 minutes. This consultation serves to get to know each other, to reduce any fears, to clarify any questions and to inform the new patient about the topics and procedures of the group program and the group rules (for example listening to each other and letting the other person finish talking). Patients will also receive a folder with an overview of the group topics, the group rules and enough space for their work sheets and individual notes. Furthermore, all patients will give their written consent that they will treat all information provided by other group members confidentially. The group sessions will take place at the outpatient clinic of the Institute for Psychology at the Albert-Ludwigs-University of Freiburg. During the sessions, tea and water will be offered and a short break of 10 minutes will be included.

The STEpS group program covers the following topics: behavioral activation (two sessions), cognitive restructuring (two sessions), coping with illness, social support, self-esteem and meaning. Every session will start with an exercise of progressive muscle relaxation as described by Jacobson [40], and with everybody giving a short summary of his or her current situation. Mindfulness-based exercises (for example, leaves on a stream) will be used as a break or closing exercise, but will not be a regular feature of the group program. All sessions will end with a final round (‘How do you feel at this moment? What is your personal take home message?’). For a detailed session overview see Table 1.

**Table 1:** Overview of the STEpS group program

Session	Topic	Content
1	Behavioral activation I	- Psycho-education about the relationship between behavior and emotion: explanation of the downward mood spiral [60]
		- Selection of individual positive activities from a checklist [61] and rating of the selected activities
		- Scheduling three positive activities for the next week
2	Behavioral activation II	- Review of homework
		- Information about the effects of physical activity on depression, physical health and wellbeing
		- Brainstorming on personal health objectives and possible physical activities to reach these objectives
		- Introduction of the MoVo concept [62] and illustration of the different steps in the motivational and volitional process of physical activity. Personal elaboration of the different steps (such as building a concrete intention, generating implementation intentions and developing counter strategies for anticipated barriers)
3	Cognitive restructuring I	- Psycho-education about the relationship between thoughts and depressive mood
		- Introduction of the ABC model [63] by means of a role-playing activity
		- Practicing the categorization in A (activating event), B (beliefs) and C (consequences) using patients’ examples. Further explanation and illustration of the relevance and changeability of cognitions
4	Cognitive restructuring II	- Introduction of typical cognitive distortions and irrational beliefs [64]
		- Identification of individual maladaptive thoughts and development of alternative thoughts
5	Coping with illness	- Information about the coping concept. Psycho-education about the person and situation specificity of adaptive coping
		- Collection of disease-related stressors (such as pain, disabilities and unclear illness course). Small group discussion about helpful coping strategies concerning one stressor. Gathering the coping strategies for the different stressors
		- Group conversation about personal intentions to try other strategies
6	Social support	- Introduction to the concept of social support and its relevance concerning chronic illness
		- Reflection on the personal social relations through drawing a social atom [65], and clarification about who in the network gives social support and how
		- Group conversation about the social network and received social support (for example wishes of change)
7	Self-esteem	- Joint development of a definition of self-esteem
		- Introduction of the four pillar model of self-esteem (self-acceptance, self-confidence, social skills and social networks) [66]
		- Reflection of personal strengths and weaknesses in different aspects of the self (for example me as a wife, me as a professional)
		- Wheel of life exercise to reflect the actual and target distribution of life energy on different life domains
8	Meaning	- Introduction of the meaning concept
		- Reflection and group conversation about meaningful moments in life
		- Illustration that everyday activities (such as a job) can be done technically or meaningfully, and that meaning can be a resource for coping
		- Reflection and group conversation about ideas for more sense of meaning in life

### Outcome measures

#### Procedure of assessments

The control group will receive all questionnaires by mail. The intervention group will receive the questionnaires at baseline and at 18-week follow-up, also by mail; the questionnaires at post-treatment will be handed out together with a short group evaluation questionnaire in the last group session. For returning the questionnaires, participants will be provided with stamped and addressed envelopes. The session questionnaires for the process evaluation of the group program will be handed out after each session, and patients will be asked to complete and return them immediately after the group session.

#### Outcome measures

Table 2 shows an overview of all variables and measurements.

**Table 2:** Key variables and measurements

Variables	Measurement	T_1_	Monitoring	T_2_	T_3_
Inclusion and exclusion criteria					
Psychiatric disorders (Axis-I)	SCID-I	x	-	-	-
Somatic disorders	self-report in telephone screening	x	-	-	-
Primary outcomes					
Anxiety and depressive symptoms	HADS-D	x	-	x	x
Secondary outcomes					
Psychological distress	BSI	x	-	x	x
Current psychosocial distress	NCCN Distress Thermometer	x	-	x	x
Changes in experience and behavior since baseline	VEV-VW	-	-	x	x
Health-related quality of life	SF-12	x	-	x	x
State of self-esteem	SSES-revised	x	-	x	x
Subjective need for psychotherapy	newly developed questions	x	-	x	x
Process evaluation					
Acceptance of treatment	treatment attendance, self-report	-	x	-	-
Therapeutic processes during the group program from patients’ perspectives	BPSR-G 2000	-	x	-	-
Covariates					
Demographic variables (sex, age, family status, education, employment, and so forth)	demographic survey	x	-	-	-
Psycho-pharmacotherapy	demographic survey	x	-	-	-
Pretreatment of psychiatric disorders	demographic survey	x	-	-	-
Psychological treatment since baseline	self-report	-	-	x	x
Changes in somatic health and psycho-pharmacotherapy since baseline	self-report	-	-	x	x
Coping with a chronic disease	TSK	x	-	-	-

*T*_*1*_ baseline/pretreatment, *T*_*2*_ post-treatment/10 weeks after randomization, *T*_*3*_ follow-up/18 weeks after randomization, *BPSR-G-2000* Bern Post Session Report 2000 modified for groups, *BSI* Brief Symptom Inventory, *HADS*-*D* Hospital Anxiety and Depression Scale, German version, *SCID*-*I* Structured Clinical Interview for DSM-IV Axis I Disorders, *SF*-*12* Short Form Health Survey, *SSES*-*revised* State Self-Esteem Scale, revised version, *TSK* Trier Scales of Coping with Disease, *VEV*-*VW* revised short form of Change Questionnaire of Experience and Behavior

#### Primary outcomes

##### Anxiety and depression

Anxiety and depressive symptoms will be measured with the German version of the Hospital Anxiety and Depression Scale (HADS-D) [41]. The HADS-D has been developed for patients with somatic diseases and assesses two domains: depression and anxiety. Each subscale consists of seven items assessing the severity of anxiety and depressive symptoms within the last week on a scale from 0 to 3. The sum score for every subscale can range from 0 to 21. Subscale scores between 0 and 7 indicate no depression or anxiety, scores between 8 and 10 indicate possible depression or anxiety and scores of 11 or higher indicate clinical depression or anxiety. Both subscales are reliable (HADS-D Anxiety Scale: α = 0.83; HADS-D Depression Scale: α = 0.82) and valid measures of depression and anxiety severity [42].

#### Secondary outcomes

##### Psychological distress

To assess psychological distress we will use the German version of the Brief Symptom Inventory (BSI) [43]. The BSI consists of 53 items and covers nine symptom domains: somatization, obsession-compulsion, interpersonal sensitivity, depression, anxiety, hostility, phobic anxiety, paranoid ideation and psychoticism. Respondents rate their symptom severity within the last seven days on a five-point scale ranging from 0 (not at all) to 4 (extremely). The Global Severity Index (GSI) is the most important of the three global scores and indicates the overall distress level of the respondent. The cutoff point for psychological distress is either a GSI T-score of 63 or higher, or two subscale T-scores of 63 or higher. The BSI is a reliable (α = 0.96 for GSI) and valid measure for the symptom severity of different mental disorders [44].

##### Current psychosocial distress

To assess the current psychosocial distress level we use the first part of the German version of the NCCN Distress Thermometer (DT) [45]. Respondents have to indicate their current distress level within the last week on an 11-point-Likert scale ranging from 0 (no distress) to 10 (extreme distress). Higher scores indicate a higher distress level. The declared cutoff point for psychosocial distress is five or higher. The DT is capable of monitoring changes in psychosocial distress over time [46, 47].

##### Changes in experience and behavior in group psychotherapy or wait-list period

Changes in experience and behavior during the group psychotherapy or wait-list period will be assessed with the Change Questionnaire of Experience and Behavior (VEV-VW) [48], a revised short form of the VEV [49]. The VEV-VW is an instrument for direct measurement of changes in psychotherapy. It consists of 27 items and assesses different situations (common experience, behavior in social situations, experience and behavior in performance situations). The respondent is requested to rate the experienced changes since a given point of time, in our study since the initial consultation. The rating scale ranges from 1 (changes for the better, for example, ‘Now I see the things more optimistically’) to 7 (changes for the worse, for example, ‘Now I see the things less optimistically’). Scores can range from 27 (maximum change for the worse) to 189 (maximum change for the better). Scores above the cutoff point of 108 indicate a better mental wellbeing, and scores below 108 indicate a worse mental wellbeing.

##### Health-related quality of life

Quality of life will be measured with the German version of the Short Form Health Survey (SF-12) [50]. The SF-12 consists of 12 items and assesses eight domains: physical functioning, physical and emotional role functioning, body pain, general health, vitality, social functioning and mental health. Physical and mental health composite scores can be calculated and range from 0 to 100, with higher scores indicating a better wellbeing. Its psychometric properties are well established [51].

##### State of self-esteem

Changes in self-esteem will be measured with the revised German version of the State Self-Esteem Scale (SSES) [52]. The SSES-revised measures state of self-esteem at a given point in time, and consists of 15 items that can be assigned to the following three factors of self-esteem: performance self-esteem, social self-esteem and appearance self-esteem. Respondents have to indicate their agreement to different statements about themselves (for example, ‘I feel good about myself’) on a five-point scale ranging from 1 (not at all) to 5 (extremely). Higher scores indicate a higher self-esteem. The internal consistency is α = 0.80 for performance self-esteem and α = 0.83 for social and appearance self-esteem [52].

##### Subjective need for psychotherapy

Changes in the subjective need for psychotherapy will be assessed by five newly developed questions. Respondents indicate their agreement to different statements (for example, ‘I currently have a high need for psychotherapy’ or ‘I currently would be okay without individual psychotherapy’) on a four-point scale from 1 (disagree) to 4 (agree).

##### Psychotherapeutic process

For the evaluation of the therapeutic process in the group program patients evaluate each group session with a session questionnaire. The session questionnaire used is a modified version of the patient version of the Bern Post Session Report (BPSR 2000) [53], extended by group specific aspects. The Bern Post Session Report modified for groups (BPSR-G 2000) includes 26 items rated on a seven-point scale ranging from −3 (not at all) to +3 (exactly). The 11 scales cover session impacts (experiences of mastery, clarification, control, problem actualization and self-esteem), other common factors (therapeutic alliance and therapeutic progress), group specific factors (cohesion and alliance with group members) and global evaluation scales (session evaluation and group setting evaluation). The psychometric properties of the original BPSR 2000 are well established [53].

##### Acceptance of the treatment

In order to measure the acceptance of the group program, dropout rates will be assessed. The therapist will document treatment attendance and disturbances. After every group session patients can describe their like or dislike of the session on three open questions in the session questionnaire. Additionally, patients will be asked to evaluate the subjective relevance of the different group topics after the completion of treatment.

#### Covariates

##### Demographic measures

We will measure age, gender and socioeconomic status with standard single-item questions.

##### Coping with a chronic disease

Coping behavior will be measured with the Trier Scales of Coping with Disease (TSK) [54]. This self-report questionnaire determines five domains: rumination, social coping (seeking social integration), threat minimization (denial), seeking information and turning to religion. There are 37 items that assess the frequency of specific thoughts and actions in recent weeks that can occur in the process of coping with a disease, on a scale ranging from 1 (never) to 6 (very often). Sum scores can be calculated for every subscale and can be compared with standard values. Its psychometric properties are well established [55].

### Sample size calculation

The literature indicates moderate effect sizes for group psychotherapy in the target group: in a meta-analysis, an average pre to post effect size of *d* = 0.49 for group psychotherapy for somatic diseases was found [24]. Effect size for group treatment compared to wait-list control (over all patient groups) was also indicated as moderate [24]. Comparable studies examining group psychotherapy for chronically ill patients with comorbid depression using HADS score as the primary outcome indicate moderate to strong effect sizes: Schuster *et al*. [30] found a strong interaction effect (*d* = 0.89) from pretreatment to six months follow-up for their CBT group therapy (six sessions) for patients with coronary or orthopedic diseases and comorbid depression, anxiety or somatoform disorders in comparison to the usual treatment of the rehabilitation clinic. Hambridge *et al*. [29] found a moderate effect from pre to post-treatment (*d* = 0.57) of their CBT group program (six sessions) for patients with coronary artery disease and comorbid depression.

In order to detect a main group effect of *f* = 0.25 [56] when adjusting for baseline severity, we will need a total sample size of 128 participants. This sample size provides a power (1-β) of 0.8 and an alpha of 0.05. Sample size calculations were done with G*power 3 software [57].

### Statistical analyses

All analyses will be based on the initial treatment assignment (intention-to-treat). Missing data will be treated according to the recommendations of Hair *et al*. [58]. First, the extent of missing data will be determined; then, the level of randomness (missing at random, missing completely at random or missing not at random) will be diagnosed applying Little’s missing completely at random test and t-tests for independent variables. According to Cho and Leonhart [59], data missing completely at random is very unlikely in a rehabilitation research setting, so missing data will probably be imputed with a missing at random-capable method (for example, using multiple imputations).

We plan to use a between-group analyses of covariance (ANCOVA) with baseline scores of the outcome variables as covariates to adjust for baseline differences. With these, we compare differences in the main outcomes between the intervention and control group at post-treatment (T_2_) and follow-up (T_3_) time points. Alpha levels will be adjusted for multiple testing. Cohen’s *d* will be calculated to measure the effect sizes. All statistical analyses will be performed using IBM SPSS Statistics 20. If we fail to achieve the target sample size within the given time frame and resources of our study, we will use analyses of variance (ANOVA) instead. Due to randomization, group differences at baseline (T_1_) will be not likely. We will then test for an interaction effect between group and time from T_1_ to T_2_ and a main effect of group from T_2_ to T_3_.

Explorative analyses will be conducted to examine potential effect moderators (demographic variables and coping style, such as seeking social integration or seeking information). Interaction terms of potential moderator variables and treatment will be created and analyzed by linear regression.

## Discussion

In this study protocol we describe the study design of a randomized wait-list controlled trial that evaluates the efficacy of a newly developed CBT group program for patients with somatic diseases and comorbid depressive or adjustment disorders, which shall be applied as a first-line treatment prior to individual outpatient therapy. We expect that study participants will benefit from the group program on a clinically significant level when compared with a wait-list control group. Moreover, we will examine the acceptance of the group program and the psychotherapeutic process during group participation.

This trial has some limitations. First, we are not sure if we will achieve the sample size and the power that is needed to calculate ANCOVAs; if not, we will use ANOVAs instead. Second, all outcomes are based on self-reported data. Complementary clinician-rated outcome measures would be beneficial, but cannot be performed due to related effort. With this in mind, we paid particular attention to choosing applied self-report measurements of primary and secondary outcomes that are well validated. Third, the study is designed as a long-term investigation with three measurement points, so missing data are to be considered. Due to the fact that the study participants are motivated to begin a therapy in our outpatient clinic, response rates are expected to be rather high. Nonetheless, missing data will occur and can increase the risk of bias. In order to minimize this risk, we will handle missing data according to the latest recommendations [58, 59].

This study also has several strengths. First, the study has a strong methodology, applying a randomized study design with a wait-list control group and a pretreatment, post-treatment and 18-week follow-up measurement point. The examined intervention was developed based on empirical evidence in a four-stage process and was tested in a pilot group. Applied outcome measurements are widely used and well-validated assessments. Inclusion and exclusion criteria are well proved using SCID-I diagnoses. Even further, inclusion and exclusion criteria are defined as naturalistically as possible, allowing a large spectrum of somatic and mental comorbidities to enhance external validity and keep the study sample comparable to routine patient care.

This randomized controlled trial will help to clarify whether the developed CBT group program is an effective treatment to reduce depressive and anxiety symptoms in patients with somatic diseases and comorbid depressive or adjustment disorder. If the group program turns out to be effective, it can be implemented as a first-line treatment option prior to individual outpatient therapy for patients with somatic diseases and comorbid depressive or adjustment disorder. The study results will contribute to better outpatient care of mental disorders in patients with somatic diseases in Germany.

## Trial status

The first study participant was enrolled in September 2013. In May 2015 patient recruitment was not completed.

## Abbreviations

ANCOVA: Analysis of covariance
ANOVA: Analysis of variance
BPSR 2000: Bern Post Session Report 2000
BPSR-G-2000: Bern Post Session Report 2000 modified for groups
BSI: Brief Symptom Inventory
CBT: Cognitive behavioral therapy
CONSORT: Consolidated standards of reporting trials
DALY: Disability-adjusted life years
DSM-IV-TR: Diagnostic and Statistical Manual of Mental Disorders, fourth edition, text revision
DT: NCCN Distress Thermometer
ERIraos: Early Recognition Inventory
GSI: Global Severity Index
HADS-D: Hospital Anxiety and Depression Scale, German version
PHQ-9: Patient Health Questionnaire
SCID-I: Structured Clinical Interview for DSM-IV Axis I Disorders
SF-12: Short Form Health Survey
SSES-revised: State Self-Esteem Scale, revised version
TSK: Trier Scales of Coping with Disease
VEV-VW: Revised short form of Change Questionnaire of Experience and Behavior
